# Association between treatment response and dose of blonanserin transdermal patch in patients with acute schizophrenia: A post hoc cluster analysis based on baseline psychiatric symptoms

**DOI:** 10.1002/npr2.12490

**Published:** 2024-10-20

**Authors:** Yoshiteru Takekita, Yuji Matsumoto, Takahiro Masuda, Kazumasa Yoshida, Yosuke Koshikawa, Masaki Kato

**Affiliations:** ^1^ Department of Neuropsychiatry, Faculty of Medicine Kansai Medical University Osaka Japan; ^2^ Medical Science, Sumitomo Pharma Co., Ltd Osaka Japan; ^3^ Data Science, Sumitomo Pharma Co., Ltd Tokyo Japan

**Keywords:** antipsychotics, blonanserin transdermal patch, cluster analysis, schizophrenia, treatment response

## Abstract

**Aim:**

To explore the optimal dose of blonanserin transdermal patch (BNS‐P) based on baseline psychiatric symptomatic characteristics during acute schizophrenia.

**Methods:**

A post hoc cluster analysis was conducted using data from a 6‐week randomized, double‐blind, placebo‐controlled study of BNS‐P (40 or 80 mg/day) in acute schizophrenia. We classified patients into three clusters based on baseline psychiatric symptoms. Efficacy was assessed using the change from baseline to week 6 in the PANSS total score. Safety was assessed by the incidence of adverse events.

**Results:**

Among 577 patients, three clusters were identified, characterized by severe psychiatric (Cluster‐S; *n* = 122), predominant negative (Cluster‐N; *n* = 191), and predominant positive (Cluster‐P; *n* = 264) symptoms. In Cluster‐P, both BNS‐P 40 and 80 mg/day reduced PANSS total score significantly more than placebo (*p* = 0.036, effect size = 0.342; *p* < 0.001, effect size = 0.687, respectively). In Cluster‐S and ‐N, only BNS‐P 80 mg/day reduced PANSS total score significantly more than placebo (*p* = 0.045, effect size = 0.497; *p* = 0.034, effect size = 0.393, respectively). The effect size was greater at 80 mg/day than at 40 mg/day across all clusters. The most common treatment‐emergent adverse events were akathisia and skin‐related adverse events in all clusters.

**Conclusion:**

BNS‐P exhibited a dose‐dependent antipsychotic effect in all clusters, particularly highlighting its efficacy in patients with predominant positive symptoms, even at lower doses. These findings provide novel and valuable insights for determining BNS‐P dose tailoring to individual symptomatic characteristics in real‐world practice.

## Abstract 2

The association between treatment response and dose of blonanserin transdermal patch (BNS‐P) was assessed in acute schizophrenia through a post hoc cluster analysis of a 6‐week randomized, double‐blind, placebo‐controlled trial. We identified three clusters based on the patients' baseline psychiatric symptoms: severe psychiatric (Cluster‐S), predominant negative (Cluster‐N), and predominant positive symptoms (Cluster‐P). BNS‐P exhibited an antipsychotic effect in all clusters at 80 mg/day, particularly highlighting its efficacy in patients with predominant positive symptoms, even at a lower dose of 40 mg/day.

## Graphical abstract

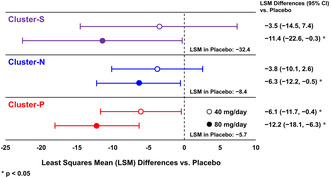

## 1 INTRODUCTION

Determining the optimal dose of antipsychotics according to individual psychiatric symptomatic characteristics during the acute phase of schizophrenia remains a significant medical need. The dose–response relationship of various antipsychotics for acute schizophrenia has been shown in a meta‐analysis.^1^ However, the optimal initial dose of antipsychotics varies depending on patient characteristics and prior use of antipsychotics.^2^, ^3^ When a patient with acute schizophrenia does not show an adequate treatment response to antipsychotic medication, it is recommended to either increase the dose of the same antipsychotic within a tolerable range or switch to another antipsychotic.^4^, ^5^, ^6^

The efficacy of antipsychotics during the acute phase of schizophrenia is different depending on the symptoms targeted for the treatment. A dose–response meta‐analysis of antipsychotics demonstrated that the treatment responses to amisulpride and olanzapine differed between patients with predominant positive and predominant negative symptoms.^1^ Another meta‐analysis found that dose–response curves for olanzapine and quetiapine extended‐release were different for positive symptoms and negative symptoms.^7^ These differences have been suggested to be due to various factors such as age, gender, and genetic background, as well as pathological conditions.^8^, ^9^

Therefore, it is challenging to develop a hypothesis for estimating the antipsychotic treatment response of individual patients. Cluster analysis is a useful analytical approach that classifies an analysis population into data‐driven homogeneous subpopulations without any specific hypothesis, allowing us to assess the antipsychotic treatment response in each subpopulation.^10^ Moreover, it can identify potential or unknown interactions and predictive factors^11^, ^12^ An analysis using this approach found that the optimal dose of asenapine differs by cluster among acute‐phase schizophrenia patients classified by differences in baseline psychiatric symptoms.^13^

Blonanserin (BNS) is an antipsychotic that has pharmacological properties with a high affinity and full antagonistic activity for dopamine D2, D3, and serotonin (5‐HT) 2A receptors.^14^, ^15^ BNS is a unique antipsychotic that has not only oral but also transdermal patch formulation. Currently, only the BNS and asenapine are available as transdermal formulations of antipsychotics worldwide. In Japan, only BNS transdermal patch (BNS‐P) has been approved for the treatment of schizophrenia, with approved doses ranging from 40 to 80 mg/day. The efficacy and safety of BNS‐P for schizophrenia have been demonstrated in a 6‐week, placebo‐controlled, double‐blind study,^16^ a 52‐week long‐term treatment study,^17^ and a meta‐analysis.^18^ However, there is no evidence on the association between the treatment response and dose of BNS‐P in patients with acute schizophrenia based on their psychiatric symptomatic characteristics. Elucidating this relationship could provide meaningful insights into the appropriate treatment approach with BNS‐P for patients with diverse background symptoms in clinical settings.

In this study, we classified the patients from the previous phase III study^16^ into three‐cluster population groups based on their baseline psychiatric symptoms and examined the association between treatment response and dose of BNS‐P in each cluster.

## 2 METHODS

### 2.1 Study design and patients

The post hoc cluster analysis was performed using data from a phase III randomized, double‐blind, multicenter, placebo‐controlled study of BNS‐P in patients with schizophrenia (ClinicalTrials.gov identifier: NCT02287584).^16^ A total of 580 patients with schizophrenia aged 18 years and older were enrolled in the original study. The main inclusion criteria included the following: an exacerbation of psychiatric symptoms <2 months before screening; the Positive and Negative Syndrome Scale (PANSS) total score ≥ 80; and a hospital stay of 2 weeks from the screening. They were randomly assigned to three treatment groups: BNS‐P 40 mg/day (*n* = 196), BNS‐P 80 mg/day (*n* = 194), and placebo (*n* = 190). They received BNS‐P 40 mg, BNS‐P 80 mg, or a placebo patch once daily. The primary endpoint was change from baseline to week 6 in PANSS total score. Each study site's Institutional Review Board/Independent Ethics Committee approved the study protocol of the original clinical trial. Written informed consent was obtained from all patients in the original clinical trial. The original study was conducted in accordance with the principles of the Declaration of Helsinki and the International Conference on Harmonization Good Clinical Practice guidelines.

### 2.2 Cluster analysis

In this analysis, 577 patients in the modified intention‐to‐treat (mITT) population of the original study (including all patients who applied ≥1 transdermal patch during the study period and who had baseline and ≥1 post baseline PANSS total scores available) were classified into three clusters using the *k*‐means method based on their baseline PANSS Lindenmayer five‐factor model scores.^19^ The preset number of clusters (three) was then validated using the sum of squared errors (SSE) within the clusters.^20^ The *k*‐means method is a representative, nonhierarchical clustering approach that selects a set of data points (*n* = *k*) as initial centroids and classifies data into homogeneous subpopulations (*n* = *k*) according to the centroids through iterative optimization.^21^, ^22^

Symptomatic characteristics were assessed in each cluster by standardized scores of baseline PANSS Lindenmayer five‐factor model scores. The standardized scores were calculated as follows:

$$\begin{array}{c} \left(\left[\text{Mean value in each cluster}\right]-\left[\text{Mean value in mITT population}\right]\right)/\\ \left[\text{Standard deviation in mITT population}\right]. \end{array}$$

### 2.3 Statistical analysis

A mixed effects model for repeated measures was used to assess the effect of each dose of BNS‐P (40 mg/day, 80 mg/day) and the placebo on the change from baseline to week 6 in PANSS total score. The model incorporated the treatment group, study visit, study site, hospitalization status, baseline PANSS total score, and interaction between the treatment group and study visit as covariates.

The effect size was calculated as the absolute least squares (LS) mean difference between BNS‐P and placebo, divided by the model‐estimated standard deviation. Treatment emergent adverse events (TEAEs), coded using the Medical Dictionary for Regulatory Activities (MedDRA) Lowest Level Term, were summarized by cluster, and the incidence rate in each dose group was compared with that in the placebo group using Fisher's exact test. Baseline patient characteristics were analyzed by comparing each dose group versus placebo in each cluster using Fisher's exact test for gender or t‐test for other parameters.

All statistical tests were conducted at a significance level of 0.05. The cluster analysis was performed using the R stats package (R Core Team and contributors worldwide). Other analyses were performed using SAS version 9.4 (SAS Institute, Inc., Cary, NC, USA).

## 3 RESULTS

### 3.1 Patient characteristics in each cluster

**FIGURE 1:**
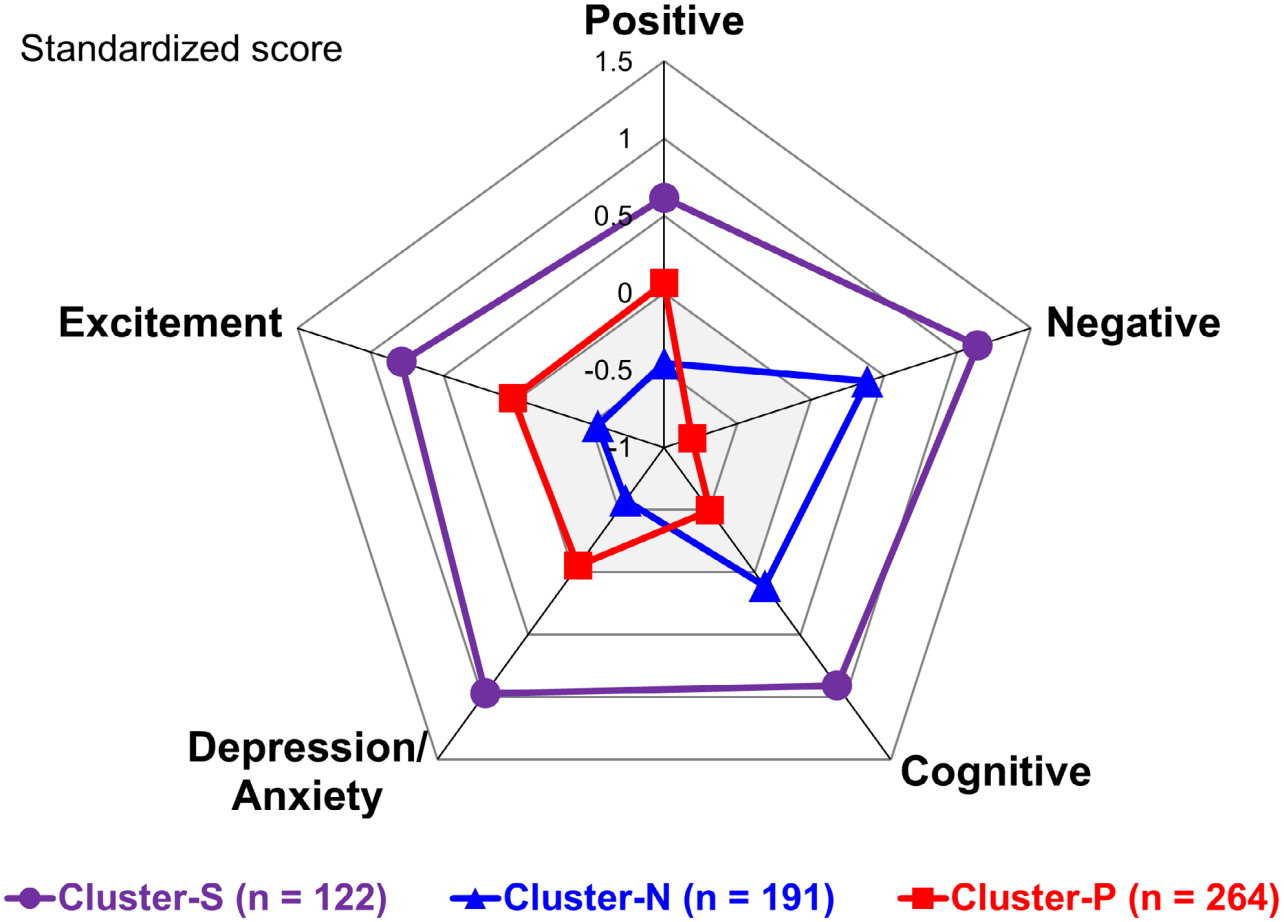
Patient classification by cluster analysis in the PANSS Lindenmayer five‐factor model. PANSS, Positive and Negative Syndrome Scale.

Cluster analysis was conducted using baseline PANSS Lindenmayer five‐factor model scores from 577 patients. The patients were classified into three clusters: Cluster‐S, with remarkably high scores on all five factors; Cluster‐N, which showed relatively higher scores on the negative and cognitive factor scales than the other three factors; and Cluster‐P, with relatively higher scores on the positive, excitement, and depression/anxiety factor scales than the other two factors (Figure 1). The number of clusters (three) was deemed appropriate based on SSE within each cluster (Figure S1). Baseline characteristics and demographics of the placebo, BNS‐P 40 mg/day, and BNS‐P 80 mg/day groups in each cluster were summarized in Table 1. There was no significant difference between the BNS‐P 40 or 80 mg/day group and the placebo group within each cluster.

**TABLE 1:** Baseline characteristics and demographics.

	Cluster‐S (*n* = 122)			Cluster‐N (*n* = 191)			Cluster‐P (*n* = 264)		
	Placebo (*n* = 36)	BNS‐P 40 mg/day (*n* = 47)	BNS‐P 80 mg/day (*n* = 39)	Placebo (*n* = 63)	BNS‐P 40 mg/day (*n* = 56)	BNS‐P 80 mg/day (*n* = 72)	Placebo (*n* = 90)	BNS‐P 40 mg/day (*n* = 93)	BNS‐P 80 mg/day (*n* = 81)
Age(years)	41.3 ± 13.9	40.9 ± 13.5	41.9 ± 18.4	42.8 ± 14.3	41.6 ± 14.9	40.9 ± 14.1	40.7 ± 13.3	40.2 ± 12.4	39.8 ± 12.4
Female	15 (41.7)	18 (38.3)	16 (41.0)	21 (33.3)	20 (35.7)	28 (38.9)	40 (44.4)	42 (45.2)	34 (42.0)
Duration of illness (years)	12.1 ± 11.8	12.7 ± 11.6	14.2 ± 13.8	15.3 ± 11.8	16.7 ± 13.4	15.3 ± 12.9	13.7 ± 12.0	12.5 ± 9.2	12.5 ± 10.8
Duration of current episode (months)	1.1 ± 0.7	1.4 ± 1.5	1.0 ± 0.6	6.3 ± 38.2	7.4 ± 46.5	1.8 ± 3.9	1.3 ± 1.5	2.7 ± 10.7	7.0 ± 49.5
PANSS total score	121.5 ± 10.0	123.6 ± 11.6	123.9 ± 12.7	96.9 ± 8.1	97.4 ± 8.4	97.6 ± 8.0	92.5 ± 8.3	93.1 ± 8.4	94.2 ± 8.7
PANSS five‐factor score									
Positive component	17.4 ± 2.6	18.2 ± 3.1	17.8 ± 3.8	14.7 ± 2.5	14.6 ± 2.6	14.2 ± 2.7	15.9 ± 3.0	16.0 ± 2.8	16.2 ± 2.7
Excitement component	14.0 ± 3.0	14.5 ± 3.8	13.4 ± 3.7	9.7 ± 2.4	9.7 ± 2.5	9.4 ± 2.6	11.1 ± 2.8	11.7 ± 2.6	11.7 ± 3.1
Cognitive component	19.6 ± 3.5	20.1 ± 3.3	20.1 ± 3.4	16.6 ± 2.5	17.3 ± 3.0	17.5 ± 3.6	14.9 ± 3.1	15.1 ± 2.8	14.7 ± 2.8
Negative component	27.0 ± 3.5	26.9 ± 4.1	28.2 ± 4.7	23.4 ± 2.6	23.4 ± 3.2	23.3 ± 2.8	17.3 ± 3.3	16.9 ± 2.7	17.6 ± 2.9
Depression/Anxiety component	18.2 ± 4.2	18.2 ± 3.6	17.5 ± 3.4	12.1 ± 3.1	12.1 ± 3.2	12.2 ± 3.0	13.9 ± 2.9	14.3 ± 3.0	14.3 ± 3.2

*Note*: Data are presented as mean ± standard deviation or as *n* (%).

Abbreviation: BNS‐P, blonanserin transdermal patch.

### 3.2 Efficacy

**FIGURE 2:**
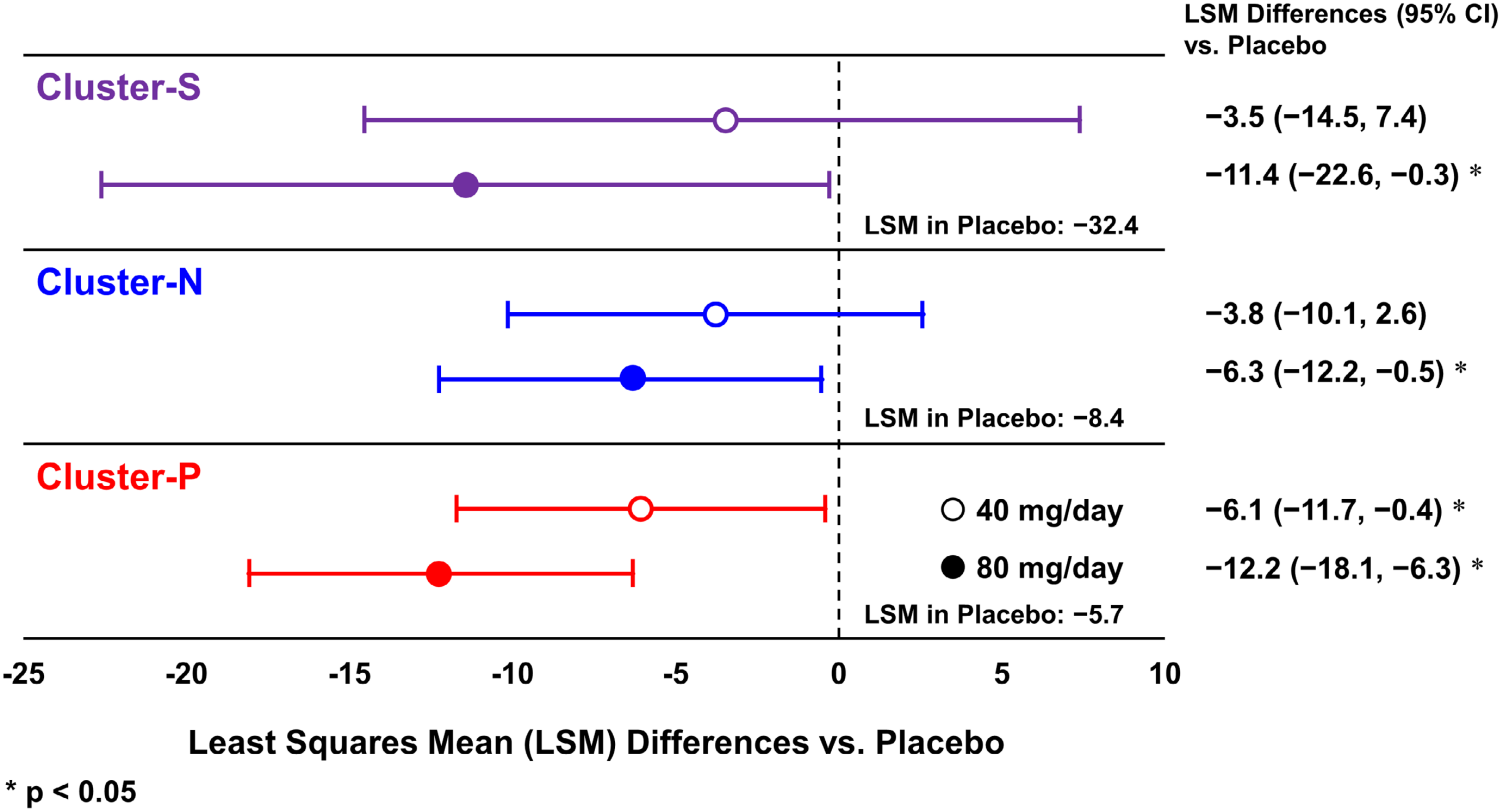
Differences in change from baseline to week 6 in PANSS total score compared to placebo. CI, confidence interval; LSM, least squares mean; PANSS, Positive and Negative Syndrome Scale.

Figure 2 shows LS mean differences in the change from baseline to week 6 in PANSS total score for the BNS‐P 40 and 80 mg/day groups versus placebo by cluster. The LS mean change in PANSS total score at week 6 in the placebo group was −32.4, −8.4, and −5.7 for Cluster‐S, ‐N, and ‐P, respectively. In Cluster‐S and Cluster‐N, the PANSS total score change was not significantly different in the 40 mg/day group compared to the placebo (Cluster‐S: *p* = 0.520; Cluster‐N: *p* = 0.239), whereas the PANSS total score was significantly more reduced in the 80 mg/day group than in the placebo (Cluster‐S: *p* = 0.045; Cluster‐N: *p* = 0.034). On the other hand, in Cluster‐P, the PANSS total score was significantly more reduced in both the 40 and 80 mg/day groups than in the placebo (40 mg/day: *p* = 0.036; 80 mg/day: *p* < 0.001). The effect sizes in the BNS‐P 40 and 80 mg/day groups were 0.154 and 0.497 in Cluster‐S, 0.239 and 0.393 in Cluster‐N, and 0.342 and 0.687 in Cluster‐P, respectively.

### 3.3 Safety

Table 2 summarizes TEAEs in the placebo, BNS‐P 40 mg/day, and BNS‐P 80 mg/day groups by cluster. TEAEs reported at a significantly higher incidence rate with BNS‐P than placebo were as follows: application site erythema (*p* = 0.030) in the 80 mg/day group of Cluster‐N; akathisia (*p* = 0.007) and application site pruritus (*p* = 0.029) in the 40 mg/day group of Cluster‐P; and akathisia (*p* < 0.001), tremor (*p* = 0.007), application site erythema (*p* = 0.014), and application site pruritus (*p* = 0.010) in the 80 mg/day group of Cluster‐P. TEAEs occurred at a significantly lower incidence with BNS‐P than placebo was schizophrenia (*p* = 0.036) in the 80 mg/day group of Cluster‐P.

**TABLE 2:** Incidence of treatment emergent adverse events by preferred term (≥5% in 40 mg/day or 80 mg/day).

	Cluster‐S (*n* = 122)			Cluster‐N (*n* = 191)			Cluster‐P (*n* = 264)		
	Placebo (*n* = 36)	BNS‐P 40 mg/day (*n* = 47)	BNS‐P 80 mg/day (*n* = 39)	Placebo (*n* = 63)	BNS‐P 40 mg/day (*n* = 56)	BNS‐P 80 mg/day (*n* = 72)	Placebo (*n* = 90)	BNS‐P 40 mg/day (*n* = 93)	BNS‐P 80 mg/day (*n* = 81)
Akathisia	1 (2.8)	3 (6.4)	1 (2.6)	1 (1.6)	0	7 (9.7)	0	8 (8.6)*	11 (13.6)*
Tremor	1 (2.8)	1 (2.1)	4 (10.3)	3 (4.8)	1 (1.8)	4 (5.6)	1 (1.1)	6 (6.5)	9 (11.1)*
Application site erythema	2 (5.6)	3 (6.4)	4 (10.3)	0	1 (1.8)	6 (8.3)*	1 (1.1)	7 (7.5)	8 (9.9)*
Application site pruritus	1 (2.8)	3 (6.4)	4 (10.3)	0	1 (1.8)	4 (5.6)	0	6 (6.5)*	6 (7.4)*
Insomnia	2 (5.6)	2 (4.3)	1 (2.6)	2 (3.2)	1 (1.8)	3 (4.2)	5 (5.6)	7 (7.5)	6 (7.4)
Constipation	0	5 (10.6)	2 (5.1)	3 (4.8)	2 (3.6)	2 (2.8)	2 (2.2)	1 (1.1)	4 (4.9)
Nasopharyngitis	2 (5.6)	1 (2.1)	2 (5.1)	3 (4.8)	1 (1.8)	4 (5.6)	3 (3.3)	3 (3.2)	3 (3.7)
Headache	2 (5.6)	3 (6.4)	1 (2.6)	0	3 (5.4)	3 (4.2)	3 (3.3)	3 (3.2)	3 (3.7)
Bradykinesia	0	0	3 (7.7)	0	0	1 (1.4)	0	1 (1.1)	2 (2.5)
Schizophrenia	1 (2.8)	3 (6.4)	0	4 (6.3)	2 (3.6)	2 (2.8)	8 (8.9)	5 (5.4)	1 (1.2)*
Psychotic disorder	2 (5.6)	3 (6.4)	1 (2.6)	1 (1.6)	4 (7.1)	2 (2.8)	3 (3.3)	1 (1.1)	1 (1.2)
Toothache	1 (2.8)	2 (4.3)	1 (2.6)	1 (1.6)	3 (5.4)	1 (1.4)	2 (2.2)	0	1 (1.2)
Dry skin	0	0	2 (5.1)	0	0	0	0	1 (1.1)	1 (1.2)
Skin abrasion	0	1 (2.1)	2 (5.1)	0	0	0	0	2 (2.2)	0
Aggression	1 (2.8)	1 (2.1)	2 (5.1)	0	1 (1.8)	0	0	1 (1.1)	0

*Note*: Data are presented as *n* (%).

Abbreviation: BNS‐P, blonanserin transdermal patch.

* *p* < 0.05 (vs. placebo in each cluster).

## 4 DISCUSSION

To the best of our knowledge, this is the first study on BNS‐P treatment for patients with schizophrenia using cluster analysis based on baseline psychiatric symptoms. We classified 577 patients from the phase III study^16^ into three distinct clusters and assessed the efficacy and safety of BNS‐P in each cluster population. The cluster analysis resulted in an apparently adequate number of patients in each cluster and revealed clear differences in symptomatic characteristics between clusters. Combined with the analysis of SSE within clusters, the findings supported the appropriateness of selecting three clusters from both statistical and clinical perspectives.

In Cluster‐S and Cluster‐N, the BNS‐P 80 mg/day group, but not the 40 mg/day group, showed a significantly greater change from baseline to week 6 in PANSS total score than the placebo group. In Cluster‐P, unlike the other two clusters, both the BNS‐P 40 and 80 mg/day groups showed significantly greater improvement in the PANSS total score compared with the placebo group. In all clusters, the effect size was greater at 80 mg/day than at 40 mg/day, suggesting that increasing the dose of BNS‐P is effective for improving psychiatric symptoms in patients with schizophrenia.

In a previous cluster analysis using a similar method to the current study,^13^ the effective dose of asenapine varied between the three clusters of patients with schizophrenia that were classified based on differences in baseline psychiatric symptoms. It is worth noting that both the previous and the present studies identified clusters with different treatment responses to antipsychotic doses, although the characteristics of baseline psychiatric symptoms classified into the clusters differed between the studies due to the data‐driven classification methods.

Positive symptoms are primarily associated with excessive activation of dopaminergic neurons via dopamine D2 receptor (D2R),^23^ and D2R inhibition by most antipsychotics is considered to contribute to improving positive symptoms. Striatal D2R occupancy by antipsychotics is a major predictive factor for treatment response in patients with positive symptoms, with 60%–65% D2R occupancy required for therapeutic efficacy.^24^, ^25^ A positron emission tomography study revealed that mean D2R occupancy by BNS‐P in human brain dose‐dependently increased and achieved the therapeutic window at both 40 and 80 mg/day,^26^ suggesting that D2Rs are sufficiently occupied in the clinical dose range of BNS‐P.

Cluster‐P was characterized by predominant positive symptoms and excitement. Thus, it is considered that the patients in Cluster‐P were highly responsive to D2R‐mediated effects among the three clusters. Given that BNS‐P can sufficiently occupy D2Rs at 40 mg/day, we consider that both 40 and 80 mg/day have shown significant efficacy only in Cluster‐P.

Negative symptoms are less likely to improve than positive symptoms through D2R inhibition.^27^ On the other hand, several studies indicated that inhibition of dopamine D3 receptor (D3R)^28^, ^29^ and serotonin 5‐HT2A receptor (5‐HT2AR)^30^ may ameliorate negative symptoms. Regarding D3R antagonist, a high level of receptor occupancy in the brain is required to improve negative symptoms.^31^, ^32^ Considering that BNS provides slightly lower D3R than D2R occupancy in the human brain,^33^, ^34^ a high dose of BNS‐P may be needed to improve negative symptoms via D3R inhibition. In addition, BNS has a high binding affinity for 5‐HT2AR, but the affinity is lower than for D2R and D3R.^15^ Although 5‐HT2AR occupancy by BNS in the human brain is unknown, it is assumed that a higher dose of BNS‐P may be beneficial for 5‐HT2AR inhibition to improve negative symptoms, considering the receptor binding affinity.

Cluster‐N was characterized by predominant negative symptoms. Thus, it is considered that the patients in Cluster‐N may require a sufficiently high contribution of D3R or 5‐HT2AR‐mediated effects, and therefore, only the high dose of BNS‐P (80 mg/day) showed significant effects. Furthermore, Cluster‐S, characterized by severe symptoms across all five factors, including positive and negative symptoms, also showed significant improvement only at the high dose, similar to Cluster‐N. The lack of significant efficacy at the low dose (40 mg/day), unlike in Cluster‐P, may be related to the severe negative symptoms in Cluster‐S. These suggest that the effects of BNS‐P via D3R and 5‐HT2AR are associated with the efficacy, especially in a population with severe negative symptoms.

TEAEs showing significantly higher incidence rates in the BNS‐P treatment groups than the placebo group in each cluster were as follows: application site erythema in Cluster‐N at 80 mg/day; application site pruritus and akathisia in Cluster‐P at both 40 and 80 mg/day; and application site erythema and tremor in Cluster‐P at 80 mg/day. Worsening of psychiatric symptoms in Cluster‐P was significantly lower with BNS‐P 80 mg/day than with placebo.

Akathisia and tremor, which are extrapyramidal symptoms (EPS)‐related TEAEs, are considered to have occurred due to D2R inhibition. The incidence rates of akathisia and tremor during BNS‐P treatment in each cluster of this study are similar to those of other second‐generation antipsychotics.^35^ A recent meta‐analysis indicated that the incidence of akathisia induced by antipsychotics increases dose‐dependently within the approved dose range.^36^ This is consistent with the findings of our study that an incidence of akathisia with BNS‐P was higher at 80 mg/day than at 40 mg/day in each cluster. Our study also showed that akathisia is less likely to occur in Cluster‐N and Cluster‐S than in Cluster‐P. This finding may be supported by the evidence that some antipsychotics do not increase the incidence of akathisia with increasing doses in patients with predominant negative symptoms.^36^ For tremor, we have not found evidence about the association between its incidence and patient baseline psychiatric symptoms. However, since both akathisia and tremor are associated with EPS, the incidence of tremors may also be affected by different baseline psychiatric symptoms, similar to akathisia.

Regarding application site skin symptoms, significantly higher incidence rates were observed in the BNS‐P groups in Cluster‐P compared to the placebo. These TEAEs were commonly observed in the BNS‐P groups in the original phase III study.^16^ To our knowledge, however, there is no evidence regarding the association between baseline psychiatric symptoms and the incidence of skin symptoms at the application site of antipsychotic patches. Thus, it is unclear which factors were associated with the relatively high incidence of these TEAEs in patients with predominant positive symptoms. One possible reason is that the sample size differences among the clusters might have affected the results; however, further investigations are required to clarify the association between predominant symptoms and TEAEs.

As described above, the effective dose of BNS‐P varied by cluster: 40 mg/day was significantly effective only in Cluster‐P, while 80 mg/day was significantly effective in all clusters. On the other hand, a higher dose was consistently associated with an increased risk of adverse events across all clusters. Thus, although 40 mg/day might be appropriate for predominant positive symptoms and 80 mg/day for predominant negative or severe symptoms in acute schizophrenia, the potential risks and benefits should be carefully considered when determining the dose in clinical practice.

This study has several limitations. First, it was based on data from a clinical trial sponsored by a pharmaceutical company. Second, it was a non‐prespecified post hoc analysis. Third, we used data from a single clinical trial for cluster analysis; therefore, the small sample size in each cluster may have impacted the statistical analysis. Fourth, since it was based on a 6‐week short‐term study, the findings do not provide insights into long‐term outcomes. Lastly, because the data analyzed were from a randomized controlled trial, there are limitations in generalizing the findings to real‐world use. Therefore, caution should be exercised when applying these findings to real‐world clinical practice. Nevertheless, since there are no other randomized controlled double‐blind trials for BNS‐P, a detailed analysis of the clinical trial data is useful to help address clinical questions. The value of this study lies in providing the first evidence on the appropriate selection of BNS‐P dose based on baseline psychiatric symptoms during the acute phase of schizophrenia. Moreover, these clinically valuable findings were obtained through a post hoc analysis without exposing patients to new invasive interventions. Further studies involving large‐scale, real‐world clinical data from diverse patients are required.

## 5 CONCLUSION

The association between treatment response and dose of BNS‐P was examined by classifying patients into three clusters based on their baseline psychiatric symptoms. Our findings suggest that BNS‐P is highly effective at low doses for patients with predominant positive symptoms and also effective at high doses for patients with severe psychiatric symptoms or predominant negative symptoms. The safety results suggest that baseline psychiatric symptoms may influence the incidence of EPS. These findings provide novel and valuable clinical insights for determining the appropriate dose of BNS‐P based on the symptomatic characteristics of each patient in real‐world practice.

## AUTHOR CONTRIBUTIONS

YT contributed to the conception and design of the study and interpretation of the results. YM and TM were involved in the study design and contributed to the interpretation of the results. KY contributed to data analysis and interpretation. YK contributed to the interpretation of the results. MK supervised the study. All authors contributed to and approved the final manuscript.

## FUNDING INFORMATION

This study including medical writing support was funded by Sumitomo Pharma Co., Ltd.

## CONFLICT OF INTEREST STATEMENT

YT has received grant funding from the Japan Society for the Promotion of Science and speaker's honoraria from Sumitomo Pharma Co., Ltd., Meiji‐Seika Pharma Co., Ltd., Janssen Pharmaceutical K.K., Otsuka Pharmaceutical Co., Ltd., Eisai Co., Ltd., MSD K.K., Daiichi‐Sankyo Co., Ltd., Pfizer Japan Inc., UCB Japan Co., Ltd., Takeda Pharmaceutical Co., Ltd., Novartis Pharma K.K., and Ono Pharmaceutical Co., Ltd. YT is also an Editorial Board member of Neuropsychopharmacology Reports and a coauthor of this article. To minimize bias, he was excluded from all editorial decision‐making related to the acceptance of this article for publication. MK has received grant funding from the Japan Society for the Promotion of Science, SENSHIN Medical Research Foundation, and Japan Research Foundation for Clinical Pharmacology and consulting fees from Sumitomo Pharma Co., Ltd., Shionogi & Co., Ltd., Otsuka Pharmaceutical Co., Ltd., Lundbeck Japan K.K., Takeda Pharmaceutical Co., Ltd.; payment or honoraria for lectures, presentations, speakers' bureaus, manuscript writing or educational events from Sumitomo Pharma Co., Ltd., Otsuka Pharmaceutical Co., Ltd., Meiji Seika Pharma Co., Ltd., Eli Lilly Japan K.K., MSD K.K., Pfizer Japan Inc., Janssen Pharmaceutical K.K., Shionogi & Co., Ltd., Mitsubishi Tanabe Pharma Corporation, Takeda Pharmaceutical Co., Ltd., Lundbeck Japan K.K., Viatris Inc., Eisai Co., Ltd., Kyowa Pharmaceutical Industry Co., Ltd., and Ono Pharmaceutical Co., Ltd. YK received speaker's honoraria from Sumitomo Pharma Co., Ltd. and Takeda Pharmaceutical Co., Ltd., and a writing fee from Lundbeck Japan K.K. YM, TM, and KY are full‐time employees of Sumitomo Pharma Co., Ltd.

## ETHICS STATEMENT

Approval of the Research Protocol by an Institutional Reviewer Board: Each study site's Institutional Review Board/Independent Ethics Committee approved the study protocol of the original clinical trial.

Informed Consent: Written informed consent was obtained from all patients in the original clinical trial. The original study was conducted in accordance with the principles of the Declaration of Helsinki and the International Conference on Harmonization Good Clinical Practice guidelines.

Registry and the Registration No. of the Study/Trial: N/A. Note that the data on which this post hoc study is based were collected in a clinical trial with clinical trial registration number NCT02287584 in ClinicalTrials.gov.

Animal Studies: N/A.

## Supporting information


Figure S1.

## Data Availability

The data supporting the findings of this study were collected through a phase III clinical trial. Due to ethical restrictions, the data cannot be made publicly available, as the disclosure of individual data was not explicitly addressed in the study protocol or the informed consent form of the clinical trial.
