# Supplementary figures and images for: Association between treatment response and dose of blonanserin transdermal patch in patients with acute schizophrenia: A post hoc cluster analysis based on baseline psychiatric symptoms
Source: Neuropsychopharmacol Rep. 2024 Oct 20;44(4):784–91. doi: 10.1002/npr2.12490 (PMC11609747; doi:10.1002/npr2.12490)

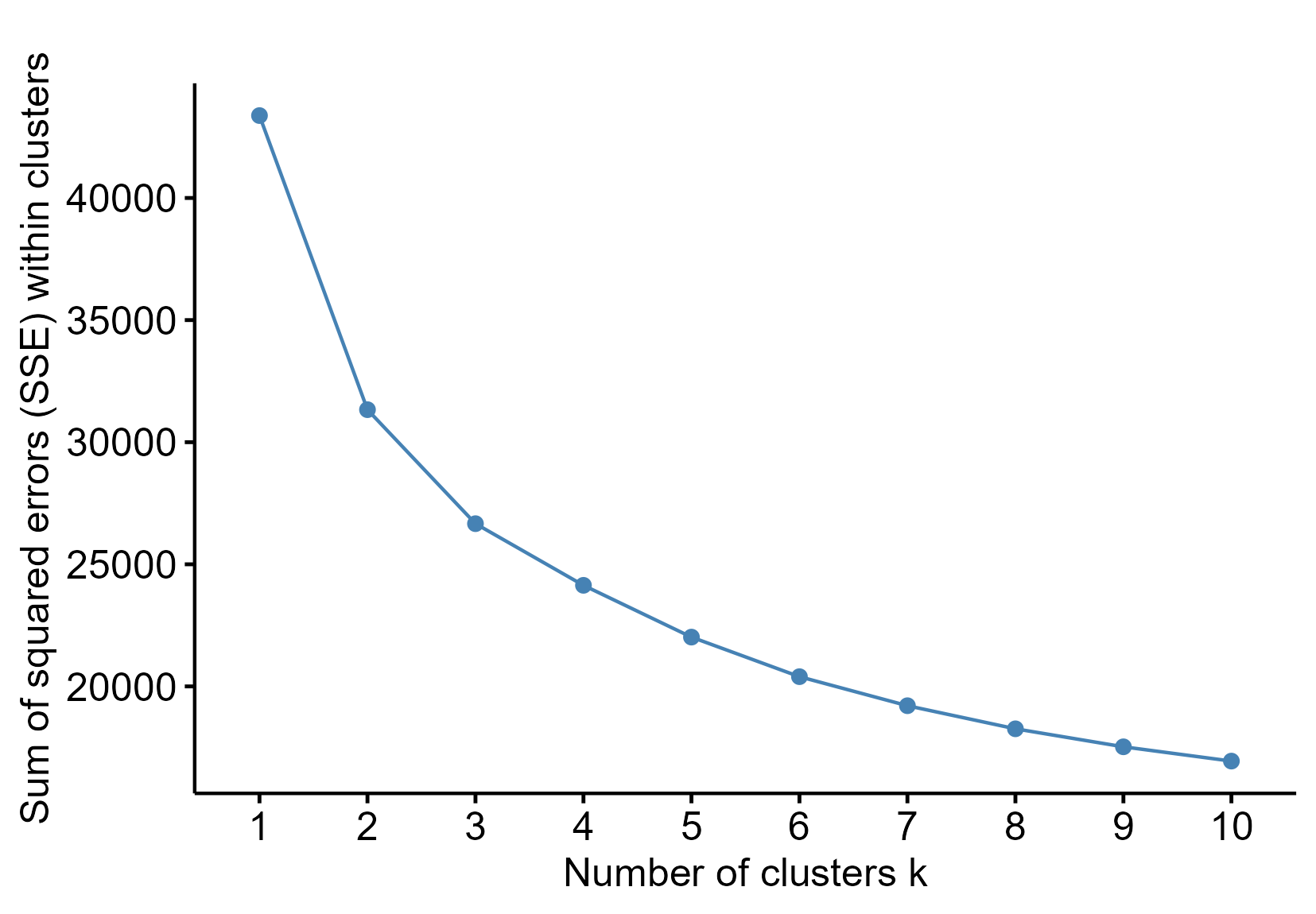

Supplement: Supplementary file 1 — Figure S1. [file NPR2-44-784-s001.tiff]
